# Editorial: Reviews in cellular neuropathology 2023: cerebral ischemia

**DOI:** 10.3389/fncel.2024.1420026

**Published:** 2024-05-02

**Authors:** Elzbieta Salinska, Creed Stary

**Affiliations:** ^1^Department of Neurochemistry, Mossakowski Medical Research Institute, Polish Academy of Sciences, Warsaw, Poland; ^2^Department of Anesthesiology, Perioperative and Pain Medicine, Stanford University School of Medicine, Stanford, CA, United States

**Keywords:** cerebral ischemia, stroke, microglia, inflammation, hypoxia tolerance

Ischemic stroke is the most common form of cerebral ischemia, and remains a leading cause of death and disability throughout the modern world. Although pre-clinical studies have highlighted several mechanisms leading to ischemia-induced neurodegeneration, the only clinical intervention to improve outcomes from ischemic stroke remains early restoration of blood flow via thrombolytics or clot retrieval, which carry several risks and contraindications. Therefore, there remains a critical need for targeted research aimed at the development of novel therapeutics. The articles collected in this Research Topic advance our current knowledge in the pathogenesis of stroke with a broad focus on post-stroke neurodegeneration and neuroinflammatory processes. In parallel, each study identifies a novel therapeutic target for stroke, opening promising avenues toward the development of new effective adjuvant stroke therapies to improve clinical outcomes in stroke survivors.

Noll et al. provide an introduction to the translational clinical hurdles of ischemic stroke and its impact on modern healthcare. Tissue plasminogen activator (tPA) remains the only pharmacologic therapy that in 1995 was approved by the FDA for the treatment of acute ischemic stroke. However, as a thrombolytic, critical side effects of tPA include bleeding and hemorrhage, underscoring a limited therapeutic window and strict regulations for its administration. The authors discuss the neuroprotective effects of other, previously tested potential stroke neuroprotective agents such as glutamate antagonists, anti-inflammatory compounds, free radical scavengers, and nerinetide (NA-1). Unfortunately, despite their promising neuroprotective effects in preclinical animal models of ischemia, these drugs failed in clinical trials. To search for new neuroprotective agents to be used in ischemic stroke therapy more effectively, the Stroke Treatment Academic Industry Roundtable (STAIR) created the preclinical study recommendations which are presented by Noll et al.. The authors present their candidate for consideration as a potential therapeutic agent. Neuregulin-1 (NRG-1), a member of the family of neuroprotective and anti-inflammatory growth factors acts through erbB tyrosine receptors activating a variety of signal transduction pathways. Notably, NRG-1 reduced ischemia-induced neuronal death and inflammation in animal models by 90% and improved the brain-blood barrier (BBB) integrity. The clinical trials in human patients with congestive heart failure demonstrate the safety and efficacy of recombinant human NRG-1. Noll et al. therefore recommend NRG-1 as an agent with outstanding potential in clinical treatment for stroke.

The initialization of fatal events leading to BBB damage and further inflammation development is discussed by Delgardo et al.. The ischemia-evoked activation of the kinin-kallikrein system leads to the formation of bradykinin, known for its pro-inflammatory and vasodilatory actions. During ischemic conditions, bradykinin binds to bradykinin one and bradykinin two receptors (B1R and B2R) and results in an increase in intracellular calcium, which then leads to downregulation of claudin-5, the tight junction protein responsible for BBB integration. This allows macromolecules to pass through the disrupted BBB, thereby resulting in brain edema. Additionally, the binding of bradykinin to its receptors attracts reactive microglia cells to infarcted areas. The authors suggest the involvement of gC1qR, a versatile binding protein in ischemia evoked activation of kinin-kallikrein, bradykinin formation, and development of inflammation and brain edema. Scientific interest in this protein revealed connections between the role of gC1qR in bradykinin formation and the pathophysiology of various neurological diseases, including ischemic stroke. The authors suggest that targeting gC1qR may be a future therapy against post-stroke inflammation and brain edema.

Wang et al., indicate the opposite action of activated microglia in the progression of inflammatory response, which is the consequence of two phenotypes of activated cells: the pro-inflammatory M1 phenotype and the anti-inflammatory M2 phenotype. It was shown in animal models of ischemic stroke that the presence of M1 and M2 in the ischemic core and the penumbra varies over time. The dominance of M2-type observed after the initial hours after brain ischemia shifts over time to M1-type, whereby M1-type microglial activation dominates in both the penumbra and the ischemic center at later time points. The inflammation mediated by M1-type microglia resulted in neuronal death and blood-brain destruction, leading to tissue damage and functional aggravation. The M1 and M2 phenotypic transformation can be regulated by transcription regulators, cytokines, and inflammatory signaling pathways. Wang et al. postulate that the exploration of mechanisms of microglia activation and regulation of phenotypical transformation can identify potential therapeutic targets for ischemic stroke treatment. The authors indicate potential candidates such as peroxisome proliferation-activated receptor γ (PPARγ), interferon regulatory factor (IRF), toll-like receptor 4 (TLR4), galectins, cysteine leukotriene receptor (CysLTR), nuclear factor erythroid 2-related factor 2 (NRF2), interleukin 4 (IL-4), signaling transducer and activator of transcription (STAT), triggering receptor expressed on myeloid cells-2 (TREM2), and cathepsin—all involved in the regulation of microglia polarization.

Most preclinical research on ischemia uses the rodent middle cerebral artery occlusion (MCAO), which closely mimics human ischemic stroke. The results obtained from these experimental stroke models are limited by variability in infarct size and animals' survival, highly dependent on the skill of the performing surgeon. Therefore, alternative models are needed. Quadros-Mennella et al. present an unexpected candidate for the search for new therapies in ischemic stroke. *Drosophila melanogaster* is a commonly used model in neuroscience due to the similarities in their centralized brain and neurotransmitter system with mammals. The authors present studies confirming mechanisms shared with mammals and discuss the recent findings explaining *Drosophila* hypoxic tolerance and how these findings may help develop new therapy for brain ischemia. An overview of compounds mediating the *Drosophila* response to hypoxia shared with mammals includes hypoxia-inducible factors (HIFs), NF-κB, Wnt, Hippo pathway, and Notch. Moreover, pathways involved in oxidative stress and insulin signaling have similar effects in *Drosophila* and mammals. According to the authors, *Drosophila melanogaster* is a promising model to study hypoxia in terms of the search for new ischemic stroke therapies. Especially alternations in NF-κB and Hippo to tolerance hypoxia look promising.

As editors, it is our hope that this Research Topic of articles will contribute to expanding knowledge about the mechanisms of neurodegeneration triggered by stroke, and ultimately inspire further research that will enable the discovery of novel effective stroke therapies that can be clinically applied in the near future.

## Author contributions

ES: Writing – original draft, Writing – review & editing. CS: Writing – original draft, Writing – review & editing.

